# A commentary on ‘Clinical efficacy and biomarker analysis of neoadjuvant camrelizumab plus chemotherapy for early-stage triple-negative breast cancer: an experimental single-arm phase II clinical trial pilot study’

**DOI:** 10.1097/JS9.0000000000001253

**Published:** 2024-03-04

**Authors:** Lei Zhang, Guannan Ma, Yanfang Jiang

**Affiliations:** a Medical Research Center, Key Laboratory of Digital Technology in Medical Diagnostics of Zhejiang Province, Hangzhou; b Genetic Diagnosis Center, The First Hospital of Jilin University, Changchun, China

*Dear Editor*,

Even when detected in its early stages, triple-negative breast cancer (TNBC), which makes up 10–15% of all breast cancer diagnoses, is an aggressive histological subtype with a significant risk of distant metastatic recurrence and death^1^. Zheng *et al*.^2^ conducted an open-label, single-arm, phase II trial on patients with triple-negative breast cancer. Research indicates that when used with nonplatinum-based chemotherapy, camrelizumab has a good partial response rate (pCR) and a manageable safety profile in patients with early-stage TNBC.

The study collected data from June 2020 to August 2021 on 23 TNBC patients. Twenty individuals were eventually operated on, and the pathological reactions were assessed. The overall pCR rate of 65% (13/20) and the bpCR rate of 70% (14/20) were achieved during surgery. Fourteen (60.9%) individuals experienced grade ≥3 treatment-related adverse events (AEs), with neutropenia accounting for 65.2% of cases. The findings shed light on the Tumor Immune Microenvironment (TIME) in pCR and non-pCR samples before and after the NAT. In pre-NAT samples, gene expression analysis revealed a greater immune infiltration in pCR patients compared to non-pCR patients. TAP1 and IRF4 were found to be prospective predictive biomarkers for NAT responsiveness by building a predictive model for NAT efficacy. Before the NAT, GSEA showed that the hypoxia and glycolysis pathways in non-pCR patients were considerably active, and this hypoxia worsened during the NAT.

But there remained a great deal of limitations. First, the main limitation of this study is the lack of a randomized control group^3^. Second, the limitation is the small sample size, which reduces statistical power, as well as the loss of follow-up, which may result in bias. Two (8.7%) patients stopped their medication during the NAT period because of intolerable adverse events, and one (4.3%) patient was excluded for noncompliance. Third, patient’s characteristics may have influenced the final result. To determine the optimal course of treatment, further in-depth prospective research, clinical trials, and analysis of indications and risk factors in different patient subgroups are needed. When estimating treatment effects based on characteristics that were different at baseline in the two cohorts using observational data, it is preferable to use propensity score matching to minimize or completely eliminate the effects of confounding.

This study explores the safety and effectiveness of using camrelizumab in conjunction with platinum-free chemotherapy as the NAT in patients with early-stage TNBC. It also assesses the predictive power of immune-related gene expressions in the response to chemotherapy. The implications of this problem for clinical practice are profound. The paucity of targeted, patient-specific medicines available in the clinic limits the personalized treatment options available to patients with TNBC^4^. Precision medicine’s future lies in utilizing multiomic techniques to direct clinical trial design, therapy, and diagnosis. A personalized treatment plan for every patient that takes into account thorough molecular profiling will make it possible to match patients to targeted clinical trials and, eventually, improve a doctor’s capacity to match treatments to malignancies^5^. Research utilizing these datasets may clarify mechanisms of resistance and response, which may be used to patient selection for treatment approaches and the discovery of more potent approaches for non-responders.

## Ethical approval

This manuscript is a comment. Do not need ethical approval.

## Consent

This manuscript is a comment. Do not need patients consent.

## Sources of funding

This work was supported by the Fund of Beijing Medical Award Foundation (No.YXJL-2021-1097-0645) and Key Research and Development Program of JianBing LingYan of Zhejiang Province (No.2024C04046).

## Author contribution

L.Z.: study concept or design, data collection, data analysis or interpretation, and writing the paper; G.M.: data analysis or interpretation; Y.J.: study concept or design, writing and revise the paper.

## Conflicts of interest

This manuscript is a comment without conflicts of interest.

## Research registration unique identifying number (UIN)

This manuscript is a comment. Do not need UIN.

## Guarantor

Yanfang Jiang.

## Data availability statement

This manuscript is a comment. Do not need data availability statement. But all the data during the current study are publicly available.

## Provenance and peer review

This manuscript is a comment without being invited.
